# Elevated Interleukin-32 Expression Is Associated with *Helicobacter pylori*-Related Gastritis

**DOI:** 10.1371/journal.pone.0088270

**Published:** 2014-03-14

**Authors:** Liu-sheng Peng, Yuan Zhuang, Wen-hua Li, Yuan-yuan Zhou, Ting-ting Wang, Na Chen, Ping Cheng, Bo-sheng Li, Hong Guo, Shi-ming Yang, Wei-san Chen, Quan-ming Zou

**Affiliations:** 1 National Engineering Research Center of Immunological Products, Department of Microbiology and Biochemical Pharmacy, College of Pharmacy, Third Military Medical University, Chongqing, PR China; 2 Department of Gastroenterology, Xinqiao Hospital, Third Military Medical University, Chongqing, PR China; 3 School of Molecular Science, La Trobe University, Bundoora, Victoria, Australia; Institut Pasteur Paris, France

## Abstract

**Background:**

Interleukin-32 (IL-32) is a recently discovered proinflammatory cytokine involved in inflammatory diseases. We investigated the expression of IL-32 and its regulation mechanism in the inflammatory response of patients with *Helicobacter pylori* (*H. pylori*) infection.

**Design and Methods:**

IL-32 mRNA and protein expression in gastric tissues was detected by quantitative real-time PCR and immunohistochemistry. The regulation of IL-32 in human gastric epithelia cell line AGS was investigated by different cytokine stimulation and different *H. pylori* strain infection.

**Results:**

Gastric IL-32 mRNA and protein expression were elevated in patients with *H. pylori* infection and positively correlated with gastritis. In *H. pylori*-infected patients, the mRNA level of IL-32 was also correlated with that of proinflammatory cytokines IL-1β and TNF-α. *In vitro* IL-1β and TNF-α could upregulate IL-32 mRNA and protein level in AGS cells, which was dependent on NF-κB signal pathway. The regulation of IL-32 expression in response to *H. pylori*-infection could be weakened by using neutralizing antibodies to block IL-1β and TNF-α. Moreover, *H. pylori*-infected AGS cells also induced IL-32 mRNA and protein expression, which was dependent on CagA.

**Conclusions:**

IL-32 level is elevated in patients with *H. pylori* infection and its expression is regulated by proinflammatory stimuli, suggesting that IL-32 may play a role in the pathogenesis of *H. pylori*-related gastritis.

## Introduction

*Helicobacter pylori* (*H. pylori*) is a Gram-negative, microaerophilic bacterium that colonizes the stomach of about 50% of the population worldwide. The persistent infection of *H. pylori* causes chronic and persistent gastritis and increases the risk of peptic ulcer and gastric cancer.

*H. pylori*-infected gastric mucosa has been characterized by the infiltration of immune cells and the production of inflammatory factors. Th1 and Th2 response are reported to mediate immune response to *H. pylori*, and Th1 response is thought to be predominant [1]. Then induction of Th17 response in *H. pylori*-infected stomach is confirmed [2]. Besides Th1, Th2 and Th17 cells, regulatory T cells (Tregs) also play an important role in *H. pylori*-related gastritis [3]. Meanwhile, proinflammtory cytokines such as IL-1β, TNF-α and IL-6 have also been shown to be elevated in *H. pylori*-infected stomach [4], and these cytokines could influence T cell immune response in inflammatory disorders, suggesting that inflammatory factors may be crucial in *H. pylori*-related gastric inflammation. However, the exact mechanism of the process has not been fully elucidated.

IL-32 is a newly identified proinflammatory cytokine produced by immune cells (NK cells, T cells, monocytes) and non-immune cells (endothelial cells, epithelial cells) [5]–[7]. It was originally cloned as a gene induced by IL-2 and called NK-4, but its function was unknown until 2005 [5], [8]. There are six splice variants including IL-32α, β, γ, δ, ε, and ζ, and diverse roles are potentially played by its different isoforms. However, a specific receptor for IL-32 has not been discovered, although neutrophil proteinase 3 binds to IL-32 with a high affinity [9]. IL-32 plays an important role in various inflammatory disorders and its expression has been shown to correlate with the severity of diseases in rheumatoid arthritis, Crohn’s disease and atopic dermatitis [10]–[12]. Moreover, IL-32 had been implied in some infectious diseases including *H. pylori* infection [13]–[16]. However, the association between IL-32 expression and *H. pylori*-induced gastritis and its exact regulation mechanism including whether auto−/paracrine effects on the expression of IL-32 may be involved in the process was remain unknown.

In the present study, we detected IL-32 expression in biopsy specimens from patients with *H. pylori* infection and analyzed the relationship between gastric IL-32 level and the severity of mucosal inflammation. Subsequently, we explored the impact of proinflammatory stimuli and *H. pylori* infection on IL-32 expression in human gastric epithelia cell lines. Our results showed that IL-32 might be involved in the pathogenesis of *H. pylori*-related gastritis.

## Materials and Methods

### Ethics Statement

Human gastric mucosal biopsies were collected by routine endoscopy at the Xinqiao Hospital of the Third Military Medical University. Blood was obtained from the same subject who underwent endoscopy for *H. pylori* serology test. The study was approved by the Ethics Committee of Xinqiao Hospital, Third Military Medical University. Written informed consent was obtained from each subject.

### Subjects

Gastric tissues and blood were collected from 54 patients (male/female = 27/27; average age 47±1.2 years) with *H. pylori* infection who underwent endoscopy at the Xinqiao Hospital of the Third Military Medical University. *H. pylori* infection was confirmed by rapid-Urease test, serology test, ^13^C-urea breath test and histology. Patients were classified as *H. pylori* positive if two of the four tests were positive. Normal gastric tissues from 47 subjects (male/female = 23/24; average age 47±1.3 years) who had negative results for all four tests were enrolled as controls.

### Biopsy Specimens and Histology Assessment

Biopsy specimens were taken from the subjects at each endoscopy. One was immediately frozen in liquid nitrogen and stored at −80°C for RNA extraction. The rest of biopsy specimens were fixed in formalin and embedded in paraffin. Haematoxylin-Eosin (H&E) stained sections were examined by two experienced histopathologist. The histological severity of gastritis was graded from normal to severe based on the density of infiltrating mononuclear and polymorphnuclear cells according to the established criteria [17], [18].

### Cell Culture

The gastric epithelial line AGS (ATCC, American Type Culture Collection) was cultured at 37°C and 5% CO_2_ in Ham’s F12 (Hyclone, Logan, UT, USA), which contained 10% fetal calf serum (FCS). AGS Cells were seeded in six-well plates at a density of 1×10^6^ cells/well and stimulated with 10 ng/ml TNF-α or/and 10 ng/ml IL-1β (PeproTech, Rocky Hill, NJ, USA); cells were collected at indicated times for analysis of IL-32 mRNA and protein expression. For the signal pathway inhibition assay, NF-κB inhibitor (BAY 11-7082), MEK1/2 inhibitor (U0126), p38/MAPK inhibitor (SB203580), JNK inhibitor (SP600125), JAK inhibitor I (all at 10 µM and all from Calbiochem, San Diego, CA, USA) or the vehicle DMSO (Sigma, Saint Louis, MO, USA) were added to the cell culture 1 hour before cytokine stimulation.

### Infection of AGS Cells with *H. pylori*

*H. pylori* 11637 strain and its isogenic CagA-negative mutant strain (CagA^-^ strain) were grown on brain-heart infusion plates containing 10% rabbit blood at 37°C under microaerophilic conditions (5% O_2_, 10% CO_2_, 85% N_2_). *H. pylori* was washed off the culture plates with PBS and centrifuged at 2500×g for 5 min, before being resuspended in PBS for optical density quantification at 600 nm (1 OD_600_ = 1×10^9^ *H. pylori*/ml). A multiplicity of infection (MOI) of 1, 10, and 100 was used for infecting AGS cells. For neutralization assays, neutralizing antibodies against TNF-α (nTNF-α, 1 µg/ml, Biolegend) or IL-1β (nIL-1β, 1 µg/ml, eBioscience) was added in the coculture system.

### RNA Isolation and Quantitative Real-time PCR

RNA was extracted from biopsy specimens or cells by TRIzol reagent® (Invitrogen, Carlsbad, CA, USA) and reverse-transcribed to cDNA using ReverTra Ace (TOYOBO, Osaka, Japan). Quantitative real-time PCR was carried out by the iQ5 Detection System (Bio-Rad, USA). The PCR primers were designed to cross an exon-intron border and used to detect the mRNA expression of IL-32, TNF-α, IL-1β and β-actin and their sequences are as follows: IL-32, forward, 5′-ACGACTTCAAAGAGGGCTACC-3′; reverse, 5′-GCCTCGGCACCGTAATCCAT-3′; TNF-α, forward, 5′-TCTCTAATCAGCCCTCTGGC-3′; reverse, 5′-ATGAGGTACAGGCCCTCTGA-3′; IL-1β, forward, 5′-GTTCTTTGAAGCTGATGGCC-3′; reverse, 5′-GTGGTCGGAGATTCGTAGCT-3′; β-actin, forward, 5′-TTCCTTCCTGGGCATGGAGTCC-3′; reverse, 5′-TGGCGTACAGGTCTTTGCGG-3′. β-actin was used as an internal control. The relative gene expression was calculated as fold change by the ΔΔCt method.

### Immunohistochemistry

Forty paraffin-embedded samples were cut into 5-µm sections. After being deparaffinized and hydrated, the sections in citrate buffer (pH = 6.0) were subjected to heat-induced antigen retrieval in a microwave oven and treated with 3% hydrogen peroxide. Following incubation with rabbit anti-human IL-32 (Abcam, MA, USA) overnight at 4°C, Slides were treated with horseradish peroxidase-conjugated secondary anti-rabbit antibody (Zhongshan Golden Bridge Biotech., Beijing, China) followed by substrate 3,3′-diaminobenzidine tetrahydrochloride (DAB). Isotype matched antibody was used as negative control. Images were acquired on a microscope equipped with a digital camera Nikon Eclipse 80i (Tokyo, Japan). For semi-quantitative analysis of the immunohistochemistry, each section was chosen for evaluating IL-32 immunostaining in gastric tissues and was graded as follows: score 0, no expression; score 1, low expression; score 2, intermediate expression; score 3, high expression.

### Western Blot

Cells were washed in ice-cold PBS and then disrupted in lysis buffer (20 mM Tris, pH 7.5, 150 mM NaCl, 1 mM EDTA, 1 mM EGTA, 1% Triton X-100, 2.5 mM sodium pyrophosphate, 1 mM glycerophosphate, 1 mM Na3VO4, 1 µg/ml leupeptin and protease inhibitor). The protein concentration was measured with a BCA protein assay kit (boster, Wuhan, China). Cell lysates were separated by 12% SDS-PAGE and transferred to a polyvinylidene difluoride membrane. The membranes were blocked for 1 h with 3% bovine serum albumin in Tris-buffered saline–Tween at room temperature and then incubated overnight at 4°C with rabbit anti-human IL-32 (Abcam, MA, USA) or mouse anti-human β-actin ((Tianjin Sungene Biotech Co., Ltd, China). Horseradish peroxidase-conjugated secondary antibody was used according to the manufacturer’s instructures. The proteins of interest were visualized by using Supersignal® West Dura Duration substrate reagent (Thermo, IL, USA).

### Statistical Analysis

All results were summarized as mean ± standard error of the mean (SEM), and statistical analysis was performed using the GraphPad Prism 5.0 Software. The differences between two groups were analyzed by the Mann-Whitney U test and multiple groups were analyzed by a one-way analysis of variance (ANOVA). When variances were detected, Spearman’s correlation was used to evaluate the degree of association between variables. P<0.05 was considered statistically significant.

## Results

### Elevated IL-32 mRNA Level Detected in Patients with *H. pylori* Infection

**Figure 1:**
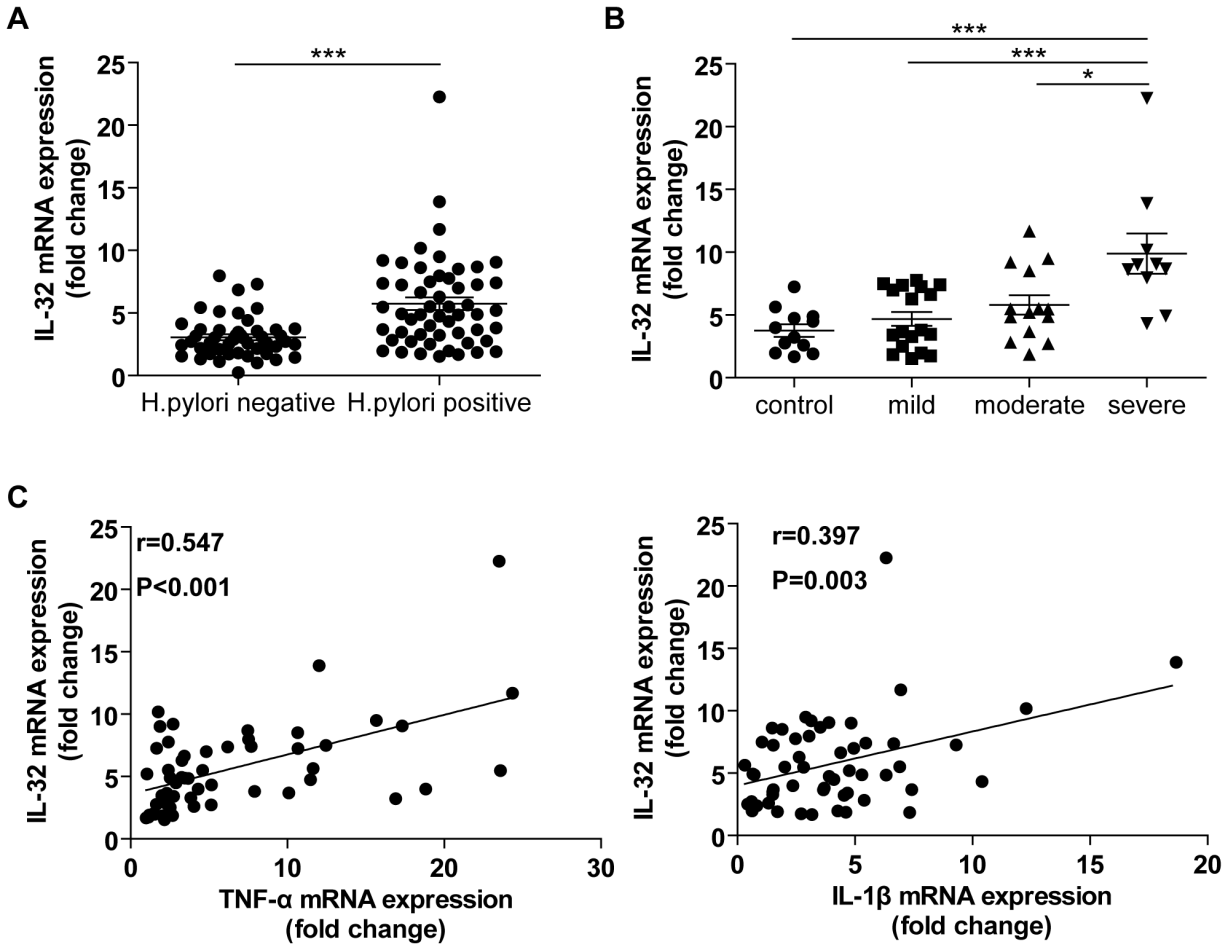
Gastric IL-32 mRNA in patients with *H. pylori* infection correlated with inflammation and inflammatory cytokine gene expression. A. IL-32 mRNA expression was detected by real-time PCR in gastric mucosa tissues from 54 *H. pylori*-positive patients and 47 *H. pylori*-negative healthy individuals; B. Analysis of the relationship between IL-32 mRNA levels and the degree of gastric inflammation in *H. pylori*-positive patients; C. IL-32 mRNA showed a positively correlation with those of TNF-α and IL-1β. *P<0.05; ***P<0.001.

To study whether IL-32 is involved in the pathogenesis of *H. pylori* induced gastritis, we first determined the IL-32 mRNA expression in gastric biopsy specimens from subjects with and without *H. pylori* infection. As shown in Fig. 1A, IL-32 expression was significantly higher in *H. pylori*-positive samples than that in *H. pylori*-negative samples (P<0.001). To evaluate whether the expression of IL-32 was related to the degree of inflammation in *H. pylori*-infected gastric samples, we divided the samples into normal and mild, moderate and severe gastritis groups based on histology assessment as described in Materials and Methods. The results showed that IL-32 mRNA levels in *H. pylori*-positive samples were positively correlated with the degree of gastric inflammation (Fig. 1B). Moreover, the level of IL-32 mRNA also showed a positive correlation with TNF-α and IL-1β mRNA levels (Fig. 1C).

### Increased IL-32 Protein Expression in Patients with *H. pylori* Infection

**Figure 2:**
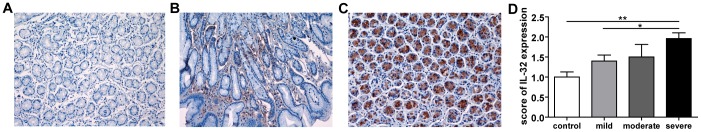
IL-32 protein was detected in the gastric biopsy tissues. A. control isotype IgG (200×); B. A representative microphotograph showing biopsy sections from *H. pylori*-negative normal controls stained for IL-32 (200×); C. A representative microphotograph showing IL-32 immunostaining in biopsy sections from *H. pylori*-positive patients (200×) ; D. The immunohistochemistry of IL-32 in the gastric biopsy tissues was semi-quantitatively graded as scores (0 = no expression; 1 = low expression; 2 = intermediate expression; 3 = high expression). *P<0.05; **P<0.01.

To visualize IL-32 expression in gastric biopsy specimens, immunohistochemical staining of IL-32 was performed on paraffin-embedded tissues. As shown in Fig. 2B, a few of IL-32-producing cells were detected in *H. pylori*–negative gastric tissues, whereas in *H. pylori*–positive gastric tissues, we observed that IL-32 protein expression was significantly increased (Fig. 2C). In addition, the semi-quantitative evaluation of IL-32 immunoreactivity confirmed that the expression of IL-32 in gastric tissues of *H. pylori*-infected patients at protein level was also significantly increased compared with those in *H. pylori*–negative gastric tissues and in mild gastritis.

### IL-32 mRNA and Protein Level were Upregulated by TNF-α and IL-1β

**Figure 3:**
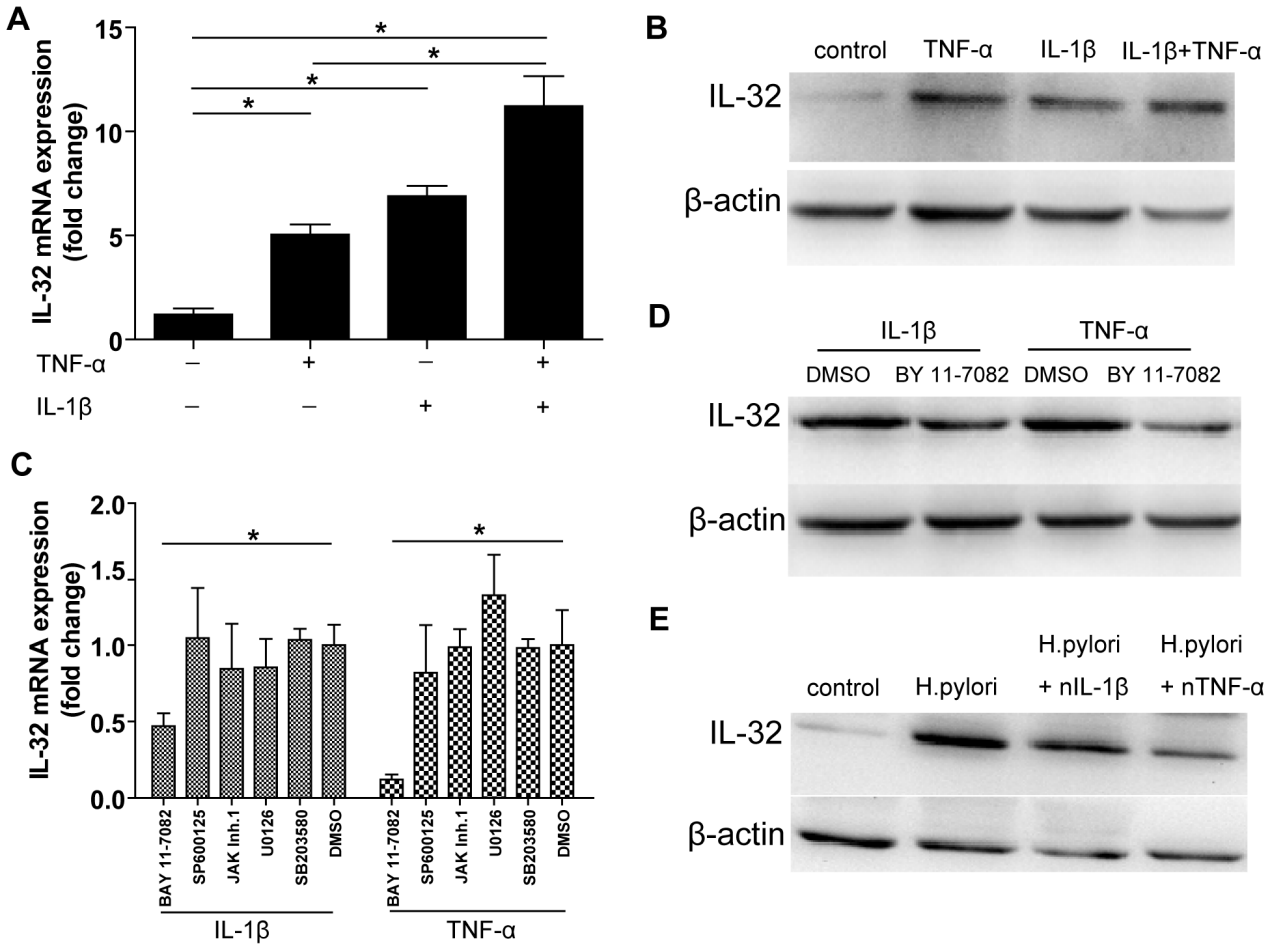
Induction and regulation of IL-32 mRNA in AGS cells. A. IL-32 mRNA expression was analyzed in AGS cells after stimulation with TNF-α and/or IL-1β for 24 hours. *P<0.05; B. IL-32 protein expression was detected by Western blot from (A); C. AGS cells were pretreated with 10 µM NF-κB inhibitor (BAY 11-7082), MEK1/2 inhibitor (U0126), p38/MAPK inhibitor (SB203580), JNK inhibitor (SP600125), JAK inhibitor I or the vehicle DMSO for 1 hour prior to IL-1β or TNF-α stimulation. IL-32 mRNA level was determined by real-time PCR. *P<0.05 versus the vehicle DMSO treated cells; D. The level of IL-32 protein was detected after AGS cells pretreation with 10 µM NF-κB inhibitor (BAY 11-7082) or not and then stimulation with TNF-α or IL-1β; E. AGS cells were infected by *H. pylori* at a MOI = 100 for 24 hours and simultaneously treated with neutralizing antibodies to block TNF-α or IL-1β.

Because we found a positive relationship between IL-32 mRNA levels and the degree of gastric inflammation in gastric tissues and correlation between IL-32 mRNA and IL-1β and TNF-α mRNAs, the regulation of proinflammatory cytokines on IL-32 mRNA expression was investigated in AGS cells. As shown in Fig. 3A, after being treated with cytokines for 24 hours, IL-32 mRNA level was significantly upregulated: 5.0±0.5-fold by TNF-α, 6.9±0.5-fold by IL-1β, and 11.2±1.4-fold by IL-1β and TNF-α. This effect was completely dependent on NF-κB signaling pathway as pretreatment with NF-κB inhibitor BAY 11-7082, but not JNK, p38/MAPK, MEK1/2 or JAK/STAT signaling inhibitors inhibited the induction of IL-32 mRNA after stimulation by TNF-α or IL-1β (Fig. 3C). Furthermore, Western blot confirmed that IL-32 protein level was also induced by TNF-α and/or IL-1β stimulation which depended on NF-κB signaling pathway (Fig. 3B and D). We next studied whether TNF-α or IL-1β was involved in *H. pylori*-induced upregulation of IL-32 expression. AGS cells were infected with *H. pylori* and simultaneously treated with neutralizing antibodies to block TNF-α or IL-1β. As shown in Fig. 3E, *H* *. pylori*-induced IL-32 protein expression was significantly decreased by using indicated cytokines-blocking antibodies.

### *H. pylori*-induced IL-32 Expression

**Figure 4:**
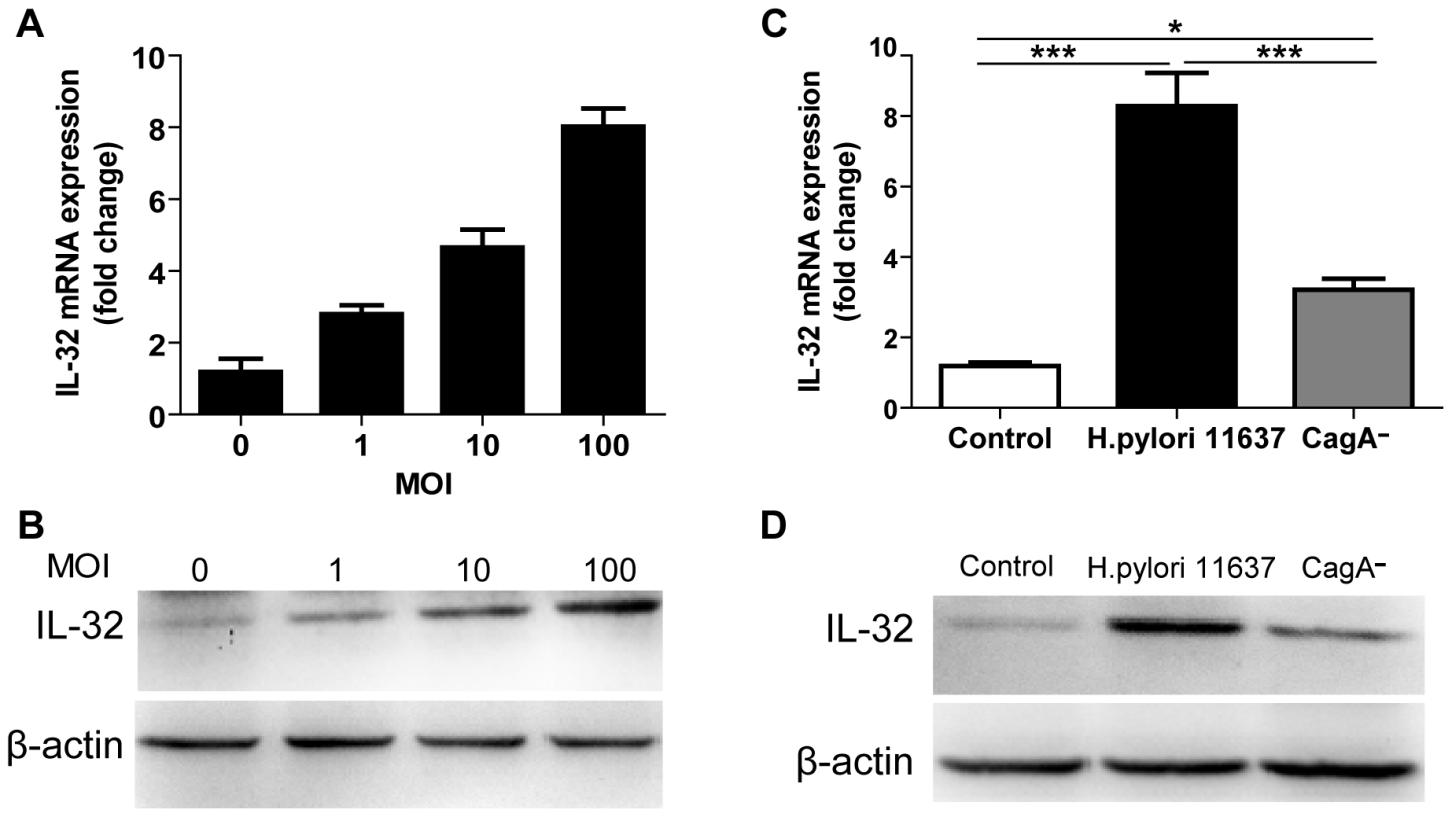
IL-32 induction in response to *H. pylori* infection. A. IL-32 mRNA level were assessed in AGS cells after *H. pylori 11637* strain infection for 24 hours at a MOI of 1, 10, 100; B. IL-32 protein level were detected in (A); C. AGS cells were stimulated with either *H. pylori 11637* strain or its isogenic CagA-negative mutant strain for 24 hours, and then the mRNA level of IL-32 was analyzed. Data are mean ± SEM of three separate experiments. *P<0.05; ***P<0.001; D. The protein level of IL-32 was detected and one representative blot was shown from (C).

To further study the direct effect of *H. pylori* on the expression of IL-32 in gastric epithelial cells, AGS cells were infected with *H. pylori* at a MOI of 1, 10, and 100. As shown in Fig. 4A and B, IL-32 mRNA and protein levels were increased after *H. pylori* infection in a dose-dependent manner. We next analyzed whether *H. pylori* strain differences would contribute to different expression of IL-32. Cells were infected with *H. pylori 11637* strain and CagA^-^ strain. Although CagA^-^ strain infection slightly increased the expression of IL-32 in AGS cells, the induction of IL-32 mRNA and protein level by CagA^-^ strain infection was significantly lower than that by *H. pylori 11637* strain infection (Fig. 4C and D).

## Discussion

In the present study, we observed that gastric IL-32 was significantly elevated in patients with *H. pylori* infection at both mRNA and protein levels. The increased IL-32 mRNA level correlated with the severity of gastric inflammation. In addition, IL-32 mRNA levels were also correlated with TNF-α and IL-1β mRNA levels in *H. pylori-*positive gastric biopsy specimens, and either of the two cytokines could upregulate the IL-32 mRNA and protein level. Furthermore, we found that *H. pylori* infection of gastric epithelial cell lines also induced IL-32 mRNA and protein expression. These results indicate that IL-32 is likely involved in gastric inflammation from patients with *H. pylori* infection.

IL-32 was recently described as a proinflammtory factor involved in many inflammatory disorders such as chronic rhinosinusitis, a condition mostly caused by gram positive bacteria infection [19], [20]. Elevated IL-32 was then reported in HCV and HBV-infected liver and to correlate with the severity of hepatic inflammation and liver fibrosis [15], [21]. In this study, we showed that IL-32 mRNA level was significantly elevated in patients with *H. pylori* infection, and a high correlation between IL-32 mRNA and gastric inflammation was also observed, suggesting that IL-32 may play an important role in *H. pylori-*infected stomach. In addition, immunohistochemistry was used to detect the source of IL-32 and results showed that IL-32 was expressed in gastric epithelial cells and its highly expression was observed in *H. pylori-*positive gastric tissues. Because IL-32 was found to induce the production of IL-8 in gastric epithelial cells and inhibited the proangiogenic factor VEGF secretion by bronchial epithelial cells [16], [22], and we also observed IL-32 mRNA level was positively correlated with IL-8 expression (data not shown). It is reasonable that IL-32 influence the function of gastric epithelial cells in *H. pylori-*infected stomach.

The classic gastric inflammation with *H. pylori* infection could be influenced by infiltrating immune cells and inflammatory cytokines, the later may include the recently discovered IL-32 [19]–[22]. In our study, we found that in vitro TNF-α and/or IL-1β stimulated AGS cells to upregulate IL-32 mRNA and protein expression, which supported a positive relationship between IL-32 and TNF-α and IL-1β in vivo. However, Th17 cytokine (IL-17A, IL-17F, IL-6), Th2 cytokine IL-4, Tregs cytokine TGF-β1 and Th1 cytokine IFN-γ all failed to induce IL-32 mRNA and protein expression in our system, although all the relevant immune cell types and cytokines were reported to infiltrate *H. pylori-*infected stomach (see Figure S1). These results suggested that IL-32 is likely produced before the infiltration of these cells, which is supported by the report that IL-32 induces dendritic cell maturation and promotes the Th1 and Th17 polarization [23]. Moreover, the induction of IL-32 mRNA and protein by TNF-α or IL-1β was blocked by NF-κB inhibitor BY 11-7082, indicating that the molecular mechanism underlying this process is likely NF-κB dependent. Interestingly, using neutralizing antibodies to block TNF-α or IL-1β in parallel with *H. pylori* infection, we observed that IL-32 protein level was significantly decreased. Therefore, our results indicated that epithelial cytokines (TNF-α and IL-1β) response to *H. pylori* infection were important for the regulation of IL-32 expression.

Our results also showed that the IL-32 mRNA and protein level were increased in a dose-dependent manner following *H. pylori* infection, suggesting that the effect of *H. pylori* infection on IL32 expression is directly physiological. In addition, CagA mutations in *H. pylori* could significantly weaken the expression of IL-32 in AGS cells, indicating that *H. pylori*–induced IL-32 upregulation was dependent on CagA. *H. pylori* express many proteins to facilitate its pathogenesis [24]. UreB is also an important virulence protein for the colonization of *H. pylori* and mediated *H. pylori*-induced dysfunction of gastric barrier [25], [26]. We found that UreB mutations also attenuated *H. pylori*–induced IL-32 mRNA expression (see Figure S2). Therefore, IL-32 expression was predominantly regulated by translocation of CagA into epithelial cells and other virulence proteins might also influence the pathogenic response of *H. pylori* to gastric epithelial cells.

In conclusion, our data demonstrated that IL-32 expression was elevated in patients with *H. pylori* infection and correlated with the severity of gastric inflammation. The regulation of IL-32 expression in response to *H. pylori* infection depends on different interacting factors. These data together suggest that IL-32 may play an important role in the pathogenesis of gastritis caused by *H. pylori* infection.

## Supporting Information

Figure S1 **AGS cells were seeded in six-well plates at a density of 1×10^6^ cells/well and stimulated with 10 ng/ml Th17 cytokine (IL-17A, IL-17F, IL-6), Th2 cytokine IL-4, Tregs cytokine TGF-β1 and Th1 cytokine IFN-γ for 24 hours, and cells were collected for analysis of IL-32 mRNA and protein expression.** Data are mean ± SEM of three separate experiments and one representative blot was shown.
(TIF)

Figure S2 ***H. pylori*** **strain 26695 were grown on brain-heart infusion plates containing 10% rabbit blood at 37°C under microaerophilic conditions (5% O_2_, 10% CO_2_, 85% N_2_), and its isogenic Urease subunit B-negative mutant strain (UreB^-^ strain) was obtained as before described** [**27**] **.** A multiplicity of infection (MOI) of 100 was used for infecting AGS and GES-1 cells. Cells were collected for analysis of IL-32 mRNA expression. Data are mean ± SEM of three separate experiments. *P<0.05; **P<0.01; ***P<0.001.
(TIF)
