# Supplementary figures and images for: Elevated Interleukin-32 Expression Is Associated with Helicobacter pylori-Related Gastritis
Source: PLoS One. 2014 Mar 14;9(3):e88270. doi: 10.1371/journal.pone.0088270 (PMC3954549; doi:10.1371/journal.pone.0088270)

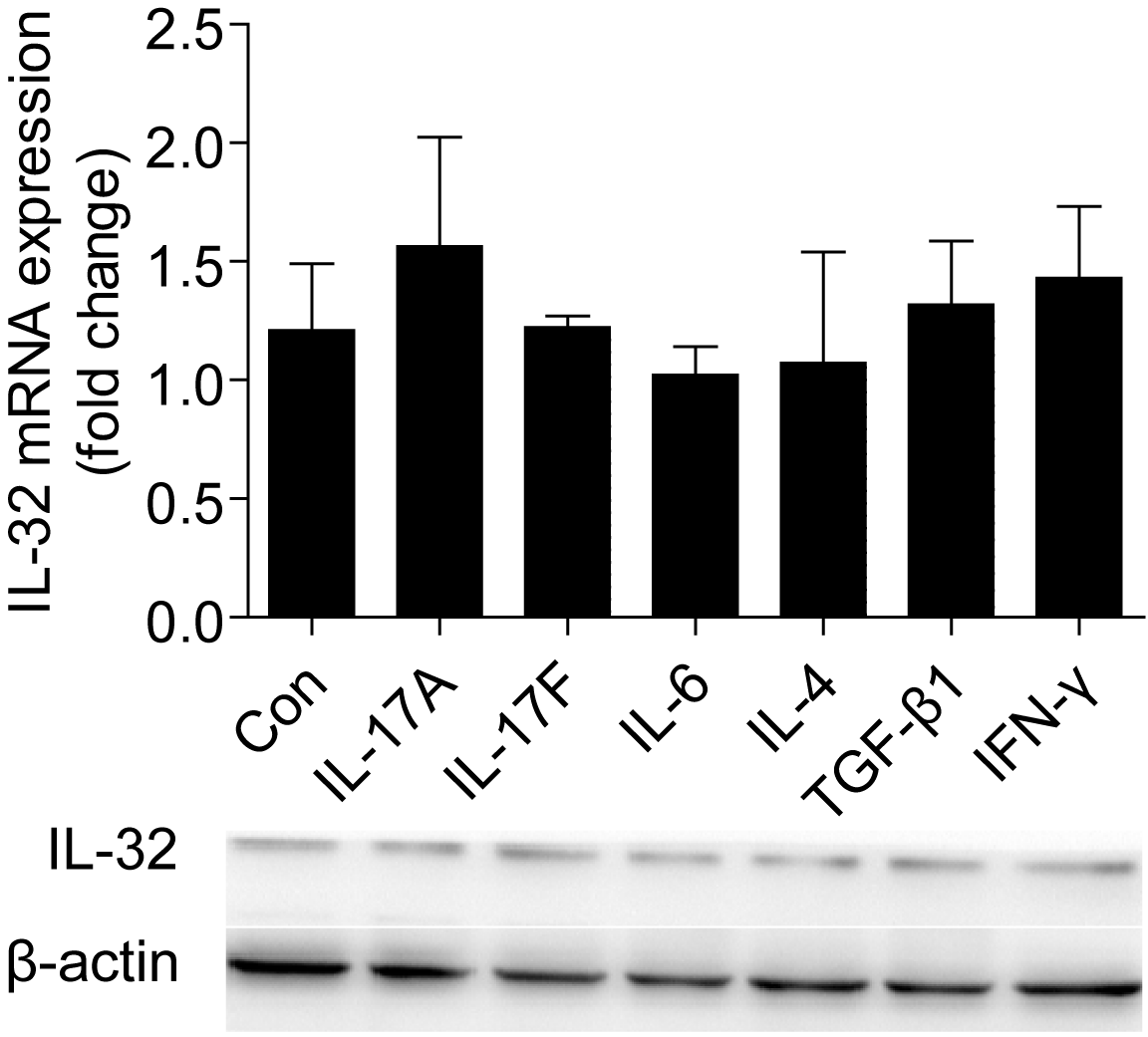

Supplement: Figure S1 — AGS cells were seeded in six-well plates at a density of 1×10^6^ cells/well and stimulated with 10 ng/ml Th17 cytokine (IL-17A, IL-17F, IL-6), Th2 cytokine IL-4, Tregs cytokine TGF-β1 and Th1 cytokine IFN-γ for 24 hours, and cells were collected for analysis of IL-32 mRNA and protein expression. Data are mean ± SEM of three separate experiments and one representative blot was shown. (TIF) [file pone.0088270.s001.tif]

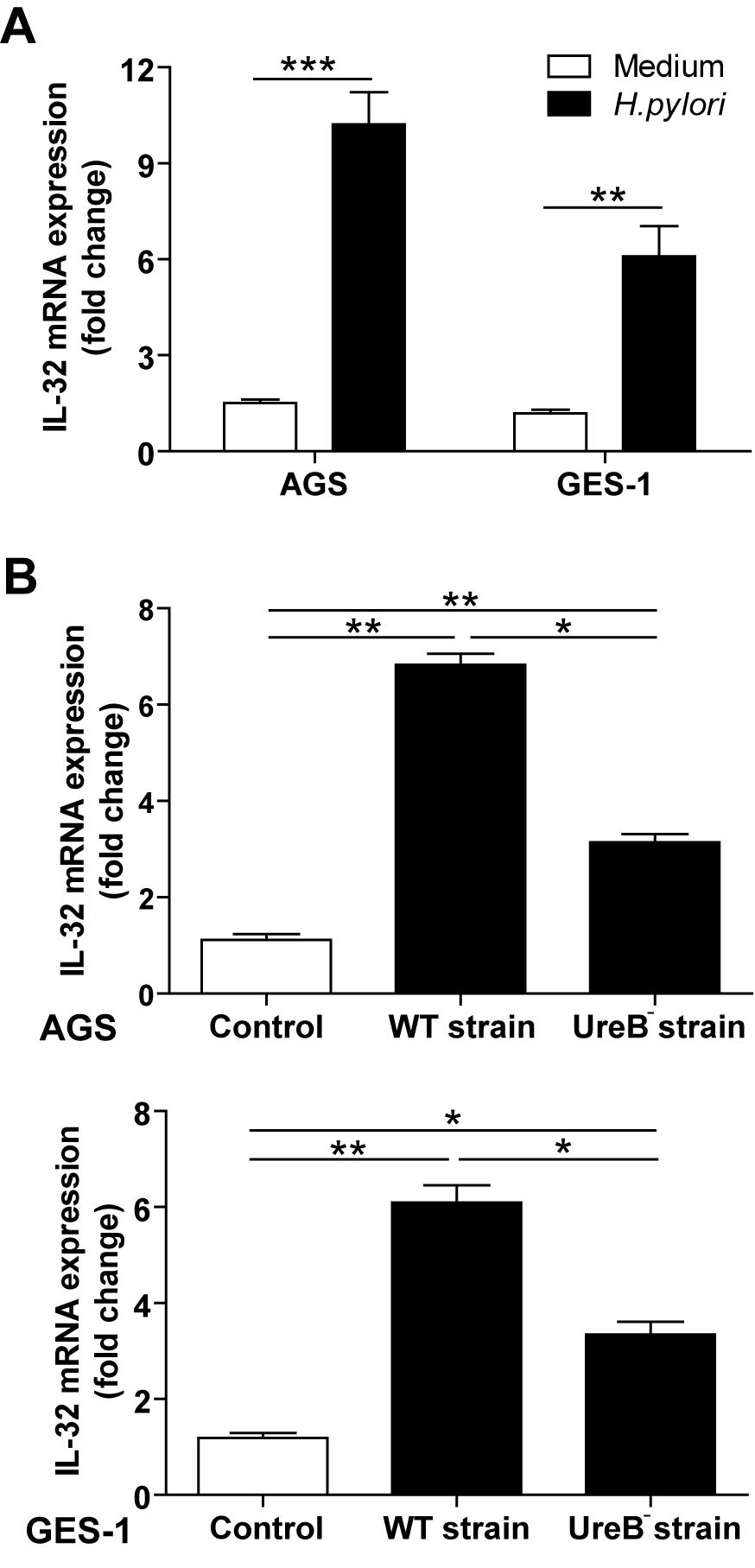

Supplement: Figure S2 — H. pylori strain 26695 were grown on brain-heart infusion plates containing 10% rabbit blood at 37°C under microaerophilic conditions (5% O2, 10% CO2, 85% N2), and its isogenic Urease subunit B-negative mutant strain (UreB- strain) was obtained as before described [27] . A multiplicity of infection (MOI) of 100 was used for infecting AGS and GES-1 cells. Cells were collected for analysis of IL-32 mRNA expression. Data are mean ± SEM of three separate experiments. *P<0.05; **P<0.01; ***P<0.001. (TIF) [file pone.0088270.s002.tif]
